# The role of feedback in improving the effectiveness of workplace based assessments: a systematic review

**DOI:** 10.1186/1472-6920-12-25

**Published:** 2012-05-02

**Authors:** Habiba Saedon, Shizalia Salleh, Arun Balakrishnan, Christopher HE Imray, Mahmud Saedon

**Affiliations:** 1 Department of Ophthalmology, Heart of England NHS Foundation Trust, Birmingham, UK; 2 Department of Otolaryngology, University Hospitals Coventry and Warwickshire, Coventry, UK; 3 Department of Vascular Surgery, Freeman Hospital, Newcastle upon Tyne, UK; 4 Department of Surgery, University Hospitals Coventry and Warwickshire and Warwick Medical School, University of Warwick, Coventry, UK; 5 Warwick Medical School, University of Warwick, Coventry, UK

## Abstract

**Background:**

With recent emphasis placed on workplace based assessment (WBA) as a method of formative performance assessment, there is limited evidence in the current literature regarding the role of feedback in improving the effectiveness of WBA. The aim of this systematic review was to elucidate the impact of feedback on the effectiveness of WBA in postgraduate medical training.

**Methods:**

Searches were conducted using the following bibliographic databases to identify original published studies related to WBA and the role of feedback: Medline (1950-December 2010), Embase (1980-December 2010) and Journals@Ovid (English language only, 1996-December 2010). Studies which attempted to evaluate the role of feedback in WBA involving postgraduate doctors were included.

**Results:**

15 identified studies met the inclusion criteria and minimum quality threshold. They were heterogeneous in methodological design. 7 studies focused on multi source feedback, 3 studies were based on mini-clinical evaluation exercise, 2 looked at procedural based assessment, one study looked at workplace based assessments in general and 2 studies looked at a combination of 3 to 6 workplace based assessments. 7 studies originated from the United Kingdom. Others were from Canada, the United States and New Zealand. Study populations were doctors in various grades of training from a wide range of specialties including general practice, general medicine, general surgery, dermatology, paediatrics and anaesthetics. All studies were prospective in design, and non-comparative descriptive or observational studies using a variety of methods including questionnaires, one to one interviews and focus groups.

**Conclusions:**

The evidence base contains few high quality conclusive studies and more studies are required to provide further evidence for the effect of feedback from workplace based assessment on subsequent performance. There is, however, good evidence that if well implemented, feedback from workplace based assessments, particularly multisource feedback, leads to a perceived positive effect on practice.

## Background

Feedback in clinical education has been defined as “specific information about the comparison between a trainee’s observed performance and a standard, given with the intent to improve the trainee’s performance” [1]. It has been suggested that the provision of feedback from formative assessments leads to a positive impact on doctors’ learning and performance [2].

Recent reforms in postgraduate medical education have brought about a greater emphasis on competency based training which focuses on outcomes rather than processes of learning. Workplace based assessment (WBA) is a system whereby doctors are assessed on clinical skills and other attributes in the context of his or her working environment. Various methods are used to provide this information including mini-clinical evaluation exercise (mini-CEX), case-based discussion (CBD), direct observation of procedural skills (DOPS), procedure-based assessment (PBA), objective structured assessment of technical skills (OSATS) and multi-source feedback (MSF). Feedback and scoring are given by the assessor and this information is compiled and fed back to educational supervisors.

Although there is considerable emphasis placed on WBA as a method of formative performance assessment, there is limited evidence in the current literature regarding the effectiveness of WBA in changing the behaviour of doctors and improving their performance. A recent literature review set out to explore the impact of WBA on doctors' education and performance [3]. The authors found that multisource feedback can lead to performance improvement although other factors have a major impact upon the response. There is a dearth of evidence about the outcome and use of feedback for continued learning and improvement. Anecdotally, trainees perceive feedback as the most useful aspect of WBA and believe that greater emphasis on the feedback component of WBA will improve its effectiveness as a formative assessment tool, hence improving trainees’ performance. The aim of this systematic review was to elucidate the impact of feedback on the effectiveness of WBAs in postgraduate medical training.

## Methods

### Search strategy

Searches were conducted using the following bibliographic databases to identify original published studies related to WBA and the role of feedback: Medline (1950-December 2010), Embase (1980-December 2010) and Journals@Ovid (English language only, 1996-December 2010). The search terms used were “feedback”, “workplace based assessment”, “direct observation of procedural skills”, “mini clinical evaluation exercise”, “case based discussion”, “multisource feedback”, “procedure-based assessment,” “objective structured assessment of technical skills”, “training” and “medical education”. In addition, hand searches using reference lists and bibliographies of included studies and review articles were performed.

### Inclusion and exclusion criteria

Studies which attempted to evaluate the role of feedback in WBA (multi-source feedback, direct observation of procedural skills, mini-clinical evaluation exercise, procedure based assessment, objective structured assessment of technical skills or case-based discussion) involving postgraduate doctors were included. Both quantitative and qualitative studies were included. Non-English literature, case reports, review articles, ‘grey literature’ (non-refereed journals and conference proceedings), commentaries, letters, editorials and studies which only included undergraduate medical students and other health care professionals were excluded. The methodological quality of the selected studies was assessed according criteria developed by Colthart et al (Table 1) [4]. Only studies where conclusions were supported by the evidence presented (grades 3 and above) were considered. All studied were initially reviewed by two reviewers (HS, MS) and summaries of the findings were derived after discussion among other reviewers (SS, AB and CHEI).

**Table 1:** **Gradings of Strength of Findings of the Paper [**[4]**]**

**Grade**	**Strength of Findings**
1	No clear conclusions can be drawn.
2	Results ambiguous, but there appears to be a trend.
3	Conclusions can probably be based on the results.
4	Results are clear and very likely to be true.
5	Results are unequivocal

### Data extraction

Two members of the review team (HS, MS) independently assessed the titles and abstracts of all identified citations. Reviewers were not masked to journal or author name because previous work has shown that this does not make a significant difference to the results of data extraction [5]. Data were extracted using a proforma including details of the research question, number of subjects, study design, setting, findings and limitations. Decisions of the two reviewers were recorded and then compared. Any disagreements were resolved by consensus with close attention to the inclusion/exclusion criteria. Other members of the review team evaluated the full text of the remaining potentially eligible papers and made a decision whether to definitely include or exclude each study according to the inclusion and exclusion criteria specified above. Data were synthesized using Kirkpatrick's four levels of educational outcomes and strength of findings (Table 2). Findings were grouped by type of intervention and described according to levels of outcome.

**Table 2:** **The Kirkpatrick (1967) model of education outcomes [**[6]**]**

**Level**	**Outcome**
1	Learners’ reactions
2	Learning of skills and knowledge
3	Changes in learner behaviour
4	Wider changes in the delivery of care

### Evidence synthesis

A statistical synthesis of the evidence was not conducted because no randomised trials involving feedback in formative assessments were identified and the prospective and retrospective studies included a variety of methods of assessment.

## Results

### Search results

Our initial search using the Ovid database yielded 3486 citations. Of these, 75 were found to be relevant articles. Following further screening of the title and abstract 41 were excluded. The remaining 34 articles were scrutinised and 12 studies fulfilled the inclusion criteria. Further searches of the Medline and Embase databases led to an additional 3 studies being identified and included. Manual searching of reference lists did not identify any additional articles resulting in a total of 15 studies, summarized in Table 3.

**Table 3:** Summary of studies included in the review

**Author**	**Study design**	**Data collection methods**	**Number**	**Country** **of origin**	**Profession**	**Aim of study (implied/stated)**	**Type of WBA**	**Grade of Strength of findings and main findings**
Archer et al [7]	Survey	Analysis of MSF data	4770	United Kingdom	Paediatrics	To report the evidence for and challenges to the validity of Sheffield Peer Review Assessment Tool (SPRAT) with paediatric specialist trainees across the UK as part of Royal College of Paediatrics and Child Health workplace based assessment programme.	SPRAT (MSF)	Grade 3. Assessor seniority is important. Free text boxes allow feedback for personal development
Bullock et al [8]	Survey	Analysis of MSF data	1928	United Kingdom	Junior doctors	To address differences in staff groups in their assessment of junior doctors’ professional attitudes and behaviour.	TAB (MSF)	Grade 3. Peers and administrators were less likely to indicate concern compared to consultants and senior nurses.
Burford et al [9]	Survey	Questionnaire	901	United Kingdom	Junior doctors and trainers	To compare perceptions of two tools for giving MSF to UK junior doctors, based on usability, usefulness and validity.	MSF	Grade 3. Trainees were asked in detail whether they would change their behaviour. Attitudes towards MSF in principle were positive and tools felt to be usable. Text-oriented tool rated more useful for giving feedback on communication and attitude
Canavan et al [10]	Survey	Questionnaire	970	United States	Five medical and one surgical specialty	To assess qualitatively written comments on multisource assessments based on psychological feedback theory for professional development	MSF	Grade 3. Quality of written feedback varies; a substantial portion of comments were useless and at worst detrimental to progress
Violato et al [11]	Longitudinal comparative study	Forms analysed on two occasions, 5 years apart	250	Canada	General Practice	Examining the validity and reliability of MSF for general practice and whether it has led to change in performance when reassessed in 5 years	MSF	Grade 4. There is evidence for the construct validity of the instruments and stability over time
Sargeant et al [12]	Qualitative study	Interviews	28	Canada	General Practice	To increase understanding of the consequential validity of MSF by exploring how doctors used their feedback and the conditions influencing this use.	MSF	Grade 3.Feedback usefulness enhanced by increasing its specificity. Strong influence of direct patient feedback on doctors’ performance
Sargeant et al [13]	Observational study	Focus group	15	Canada	General Practice	Exploration of physicians’ reactions to MSF, perceptions influencing these and the acceptance and use of feedback	MSF	Grade 3. Physicians’ perceptions of the MSF process and feedback can influence how and if they use the feedback for practice improvement.
Wellor et al [14]	Observational study	Questionnaire based ratings and written answers	92	New Zealand	Anaesthetics	To evaluate mini-CEX for both summative and formative assessment for anaesthetics training	Mini CEX	Grade 3. Factors that facilitated or hindered implementation or limited effective feedback were identified
Wellor et al [15]	Survey	Analysis of mini-CEX forms	331	New Zealand	Anaesthetics	Psychometric characteristics, logistics of application, and impact on the quality of supervision of the mini- CEX	Mini CEX	Grade 3. The positive effect of the mini CEX on feedback, its relative feasibility, and acceptance as a potential assessment tool was demonstrated
Holmboe et al [16]	Observational study	Videotaping of feedback sessions	107	United States	Primary care and internal medicine	To examine how often faculty provided recommendations and used interactive techniques when providing feedback as part of a mini CEX.	Mini CEX	Programs should consider both specific training in feedback and changes to the miniCEX form to facilitate interactive feedback.
James et al [17]	Observational study	Times taken to complete the consenting and operative components of the forms were recorded.	22	United Kingdom	Surgery	Assessing the time required to complete PBA forms and ease of use in the surgical workplace.	PBA	Grade 3. PBAs are feasible in clinical practice and are valued by trainees as a means of enabling focused feedback and targeted training.
Marriot et al [18]	Prospective observational study	Direct observation using the PBA.	749	United Kingdom	Surgery	The aims were to evaluate the validity, reliability and acceptability of PBA.	PBA	Grade 3. PBA demonstrated good overall validity and acceptability, and exceptionally high reliability.
Murphy et al [19]	Prospective study	Questionnaire	171	United Kingdom	General Practice	To investigate the reliability and feasibility of six potential workplace-based assessment methods	MSF, criterion audit, patient feedback, referral letters, significant event analysis, and video analysis of consultations.	Grade 3. Two WBA tools involving patient and colleague feedback have high reliability suitable for high stakes WBA in the general practice setting.
Cohen et al [20]	Survey	Questionnaire	138	United Kingdom	Dermatology	To collate the experience and views on three workplace assessments	DOPS, mini- CEX, MSF	Grade 3.Trainees appreciate the formative benefits which derive from the assessments, namely feedback and reassurance of satisfactory performance.
Johnson et al [21]	Observational study	Questionnaires and focus groups	120	United Kingdom	Medicine	To gain feedback from trainees and supervisors in relation to components of core medical training including workplace- based assessments,	All WBA	Grade 4.WPBA assessments were well received as means of evidencing achievement and for learning development The majority of trainees felt that in particular the feedback following WBA assessments had been useful.

### General findings

The 15 identified studies which met the inclusion criteria and minimum quality threshold were heterogeneous in their methodological design. A narrative overview is therefore provided rather than a meta-analysis. A wide range of WBAs were covered in the included studies. 7 studies focused on MSF, 3 studies were based on mini-CEX, 2 looked at PBA, one study looked at WBAs in general and 2 studies looked at a combination of 3 to 6 WBAs. 7 studies originated from the United Kingdom. Others were from Canada, the United States and New Zealand. Study populations were doctors in various grades of training from a wide range of specialties including general practice, general medicine, general surgery, dermatology, paediatrics and anaesthetics. All studies were prospective in design, and non-comparative descriptive or observational studies using a variety of methods including questionnaires, one to one interviews and focus groups. They all showed a modification of skills and attitudes or behavioural or willingness of learners to apply new knowledge & skills (Kirkpatrick Levels 2 and 3) [6]. None of the studies showed an improvement in learning and performance as a direct result of WBA (Kirkpatrick Level 4).

### Specific findings

#### Multisource feedback (MSF)

MSF is believed to increase motivation among staff, translating into positive behaviour change, increased productivity and self awareness which are fundamental for the progress of any organisation [22]. A non-comparative action based study by Archer et al found that MSF in the form of the Sheffield Peer Review Assessment Tool (SPRAT) does not provide enough data on trainees about whom concerns are raised, and more assessments are required for these trainees [7]. They also felt that unregulated self-selection of assessors introduces leniency bias and that this should end. Although free-text boxes allowed comments for feedback, no clear evidence was presented to show a change in practice. In an analysis of MSF data, Bullock et al demonstrated a trend towards becoming more critical in assessing trainees as seniority increases [8]. Feedback was provided by a designated trainer after completed forms were returned unseen to a central point and they stated that remedial action is undertaken as appropriate.

A postal questionnaire to trainees and trainers showed that the perceived effectiveness of multisource feedback was low [9]. There were small but significant preferences for textual feedback, shown by the team assessment of behaviour (TAB), which has large free-text boxes, being perceived as more useful than the mini-PAT, which has a numerical scale and only a small space for comments. Elements which were more likely to be changed as a result of feedback were medical knowledge and teaching and training skills. The aspect which was least likely to change was relationships with patients. TAB was felt to be more useful on items related to communication and professionalism. The expected influence of the feedback was low, with nearly a third of trainees not anticipating to change in response to feedback. The relationship between intention to change in any area and the perceived positivity or negativity of feedback was also extremely low. Assessors based their feedback on both direct and indirect observation, in conjunction with discussion with colleagues and comments from patients and other health care professionals.

Canavan et al analysed phrases in feedback comments written by observers who completed surveys to provide developmental feedback to residents and fellows [10]. They looked at the valence of feedback (positive, negative, or neutral), its level of specificity, and whether it was behaviour based or directed toward the learner’s “self”. 74.5% of surveys contained at least one global judgement. Behaviour-oriented phrases occurred less frequently, and general behaviours were mentioned more often than specific behaviours. Negative feedback phrases were found in 10.3% surveys. Similar to the positive comments, many were self-oriented, which can lead to a decline in performance [23]. The desirable characteristics of feedback were found to be specificity, behavioural focus, and sufficient clarity to be of great potential value to trainees.

A longitudinal study investigated changes in performance for 250 doctors who participated in MSF twice, 5 years apart [11]. All the ratings increased between times 1 and 2, although the increase for patient ratings was not significant. The change in ratings by co-workers and medical colleagues were in the small-to-moderate range. The reasons for relatively little change occurring between the two time-points include the scores being high initially or that the data were not sufficiently compelling. Also, when only a few aspects of behaviour are advised to change in a survey containing more than 100 items, its effect will not be great.

A qualitative study by Sargeant et al found that doctors did not make changes if feedback from MSF was positive, and only 7 out of thirteen doctors who received negative feedback changed their behaviour [12]. The feedback most consistently used was specific, received from patients, and addressed communication skills. The feedback least frequently used addressed clinical competence and came from medical colleagues. Another qualitative study by Sargeant et al using focus group interviews found that family physicians generally agreed with their patients’ feedback [13]. However, responses to medical colleague and co-worker feedback ranged from positive to negative, and did not always result in a change in behaviour.

#### Mini-clinical evaluation exercise (Mini-CEX)

Studies on the mini-CEX in trainee anaesthetists in New Zealand showed a positive effect of feedback and a perceived very positive educational impact [14,15]. In the written feedback fields of the Mini-CEX form, 95% of specialists wrote comments under ‘things that the trainee did well’, 70% recorded comments in ‘areas for improvement’, and 60% wrote down an ‘agreed action’ [15]. Trainees felt there was not a strong culture of feedback, but that the mini-CEX facilitated feedback. Holmboe et al recorded feedback from mini-CEX sessions in a prospective observational cohort study and showed that mini-CEX frequently leads to a recommendation for improvement, with the majority of the recommendations focused on the clinical skills of medical interviewing, physical examination, and counselling [16].

#### Procedure based assessment (PBA)

James et al looked at the PBA tool in a non-comparative observational study and found that completion of the PBAs resulted in focused feedback to the trainees about their practice [17]. As a result, the trainees in this study valued this structured approach because it enabled subsequent training to be targeted appropriately. Marriot et al also studied PBA and showed that trainees reported the feedback provided by the clinical supervisor as moderately useful to very useful. Clinical supervisors rated feedback similarly [18].

### Other assessments

Murphy et al investigated 6 different instruments (criterion audit, multisource feedback, patient satisfaction ratings, assessment of referral letters, significant events analysis, and analysis of videotaped patient interactions) in General Practice registrars [19]. They highlighted the important role of feedback from patients and colleagues. A questionnaire survey of dermatology trainees collated the experience and views on MSF, DOPS and mini-CEX [20]. Trainees appreciated the formative aspects of the assessments, especially feedback, although not all trainees reported receiving useful feedback. Johnson et al’s questionnaire and focus group study of core medical trainees on their views of the curriculum and assessment found that the majority of them felt that in particular the feedback component of WBA assessments had been useful [21].

## Discussion

This systematic review aimed to evaluate the effectiveness of feedback in WBAs. The studies were all observational and there were no randomised controlled trials. The majority of the studies were seeking perceptions and self-reported changes rather than measuring actual change in practice. This is because measuring changes in practice and attributing them to feedback from the WBA is extremely difficult due to confounding factors and problems with study design. Most of the evidence to support the use of feedback from WBAs comes from studies on MSF. This may be because, whereas in other assessments the emphasis may be upon performing a procedure correctly or the management of a particular patient, MSF has the sole purpose of providing feedback of doctors’ practice and behaviours. This opportunity is often missed, as found in the study by Canavan et al which analysed comments made on MSF forms [10]. Many forms contained no comments at all and, of those that did contain comments, a significant proportion were found to lack actionable information, thus limiting their usefulness. Global judgments were more frequently used and although these may build the confidence of the person being assessed, they do not give an indication of how they should behave in order to improve their practice and future actions. Most of the trainees in the study by Burford et al did not anticipate changing their behaviour as a result of feedback from the MSF tools used, but the perceived usefulness was consistently higher with the TAB compared to the mini-PAT [9]. The greater space for free text in the former tool allows valuable information to be transmitted back to the trainee which they can use to inform a change in practice, rather than simply a numerical score.

MSF has the potential to be a useful tool but the current evidence suggests that in order for this to occur, the way in which it is used must be improved. Comments should be provided and these should be specific and action-based. Reasons why it is currently under-utilised include time constraints of an already busy clinical workload, regarding WBA as cumbersome, a lack of training on how to provide feedback and a lack of trust in the formative nature of the assessment, as learners may feel that the feedback may have a negative impact on their training [10].

Other WBAs methods such as the mini-CEX, and DOPS did not show any clear evidence of leading to a change in behaviour. The use of the mini-CEX was strongly advocated to improve feedback, but pointed out that feedback is offered less frequently than is desirable [14]. Cohen et al found that half of the dermatology trainees surveyed reported that learning points had been identified from the mini-CEX, and that feedback and learning were identified most frequently as positive aspects of the process [20]. This implies that feedback is valued and a change in behaviour may occur, but does not show this. A fifth of respondents on the mini-CEX expressed reservations about the quality of feedback; for DOPS, 14% reported that insufficient time was allowed for feedback and only 45% identified learning points arising from the process. There were no studies looking at case based discussion so the effect of this assessment on doctors’ performance is undeterminable. Further research in this area is therefore warranted.

The highest Kirkpatrick level reached by any of the studies was level 3 which indicates a change in behaviour and documents the transfer of learning to the workplace or willingness of learners to apply new knowledge and skills. Others were level 2, showing changes in the attitudes or perceptions among participant groups towards teaching and learning.

Feedback may not produce intended outcomes and may even have detrimental consequences, such as decreased motivation and reduced performance. In one study feedback perceived as being strongly negative generally evoked emotional responses, including anger and discouragement [13]. Trainers reportedly often avoid giving feedback, in order to prevent offence or provoking defensiveness [24,25]. Several studies suggested that maximizing opportunities for training of assessors in giving optimal feedback and administering assessments would improve the quality of feedback. If WBAs are simply used as a box-ticking exercise, without sufficient emphasis on feedback, then any gains will be limited [26].

### Limitations

This systematic review had some limitations. The studies were uncontrolled thereby limiting the strength of findings but this may be due to the difficulties in assessing the effect of feedback on future performance of doctors. Limitations in our methodology include the grey literature not being reviewed and only including studies in the English language which may have led to bias. Another limitation of the study is the focus on feedback which is only one potentially beneficial aspect of WBA. Others can include on the job training whilst being observed by a senior and documentation of competence in a particular area. [27]

## Conclusions

The relationship between feedback and outcome is not always straightforward and may not always achieve the desired results [28]. Good feedback can lead to increased motivation and confidence in trainees. On the other hand, negative feedback is not aimed to demotivate or demoralise a trainee, but should be taken as constructive criticism for trainees to improve. More studies are required to provide further evidence for the effect of feedback from WBAs on subsequent performance, as the evidence base contains few high quality conclusive studies. Although a difficult area to research, more randomised controlled studies on a change in behaviour following feedback from specific WBAs should be encouraged. There is, however, good evidence that if well implemented, feedback from WBAs, particularly MSF, leads to a perceived positive effect on practice.

## Competing interests

The authors declare that they have competing interests.

## Authors’ contributions

MS and HS conceived of the study, and participated in its design and coordination and drafted the manuscript. SS and AB participated in the drafting of the manuscript. CHEI revised the manuscript. All authors read and approved the final manuscript.

## Pre-publication history

The pre-publication history for this paper can be accessed here:

http://www.biomedcentral.com/1472-6920/12/25/prepub
